# 117. Trends in Four-class HIV Drug Resistance in Treatment-experienced Patients in the United States

**DOI:** 10.1093/ofid/ofab466.117

**Published:** 2021-12-04

**Authors:** Dusica Curanovic, Johnny Lai, Christos J Petropoulos, Charles M Walworth

**Affiliations:** Monogram Biosciences, San Francisco, California

## Abstract

**Background:**

Despite the availability of potent antiretroviral therapy, only 56% of people living with HIV in the US were virally suppressed in 2018. Drug resistance can hinder suppression, especially among treatment-experienced patients, in whom the prevalence of 4-class drug resistance (4CR) is unknown.

**Methods:**

Genotypic results of PhenoSense GT® *Plus* Integrase (Monogram Biosciences, South San Francisco, CA) obtained from Apr 2014 to Dec 2020 were used to assign susceptibility to nucleos(t)ide reverse transcriptase, non-nucleoside reverse transcriptase, integrase, and protease inhibitors (NRTIs, NNRTI, INIs, and PIs). Data were analyzed using summary statistics, 2 proportion Z test, one-way ANOVA and Tukey-Kramer; p < 0.05 was significant.

**Results:**

Among 13,651 patients with 15,372 tests, median age was 43 years; most had HIV-1 subtype B infection (95.09%), followed by AG (1.32%).

Among 12,303 patients with only one test, 4CR prevalence was 1.55%. Among 1,348 patients with more than one test, 4CR was seen in 3.64% of patients, and in 4.60% if cumulative resistance reports were assembled for each patient. Patients with 4CR were significantly older than those with less resistance.

The incidence of 4CR fluctuated, with a decline from 2.61% of patients tested in 2014 to 1.38% in 2017, an increase to 2.36% in 2018, and a decline to 1.56% in 2020. Among patients with more than one test, 21.01% gained resistance to a drug in a new class over an average of 19.5 months.

Most new resistance each year was to NNRTIs, followed by NRTIs, INIs, and PIs. The incidence of PI resistance declined for PIs from 13.34% of patients tested in 2014 to 11.82% in 2020, but increased for INIs from 14.56% in 2014 to 16.49% in 2020. The regimen expected to be suppressive in the greatest proportion of patients was dolutegravir + darunavir/cobicistat (94.51%).

**Conclusion:**

The prevalence of 4CR has declined over time, but remains clinically relevant, particularly in older patients who may struggle with adherence due to complex regimens, comorbidities and polypharmacy. New drug classes may benefit this group. The concurrent increase in INI and decline in PI resistance may reflect changes in prescribing practices. Drug resistance may be underestimated unless cumulative resistance is determined.

**Disclosures:**

**Dusica Curanovic, PhD**, **Monogram Biosciences** (Employee) **Johnny Lai, BS**, **Monogram Biosciences** (Employee, Shareholder) **Christos J. Petropoulos, PhD**, **Monogram Biosciences** (Employee, Shareholder) **Charles M. Walworth, MD**, **Monogram Biosciences** (Employee, Shareholder)

